# Nitric oxide-independent stimulation of soluble guanylate cyclase attenuates pulmonary fibrosis

**DOI:** 10.1186/1471-2210-11-S1-O9

**Published:** 2011-08-01

**Authors:** Oleg V Evgenov, Lin Zou, Ming Zhang, Mari Mino-Kenudson, Eugene J Mark, Emmanuel S Buys, Michael J Raher, Yan Li, Yan Feng, Rosemary C Jones, Johannes-Peter Stasch, Wei Chao

**Affiliations:** 1 Department of Anesthesia, Critical Care and Pain Medicine, Massachusetts General Hospital, Harvard Medical School, Boston, Massachusetts 02114, USA; 2 Department of Pathology, Massachusetts General Hospital, Harvard Medical School, Boston, Massachusetts 02114, USA; 3 Institute of Pharmacy, Martin Luther University, 06099 Halle, Germany; 4 Cardiovascular Research, Bayer HealthCare AG, 42119 Wuppertal, Germany

## Background

Pulmonary fibrosis (PF) is an increasing cause of morbidity and mortality with five million people affected worldwide and a median survival time of less than three years. Effective anti-fibrotic agents for PF are currently lacking. The majority of patients with PF develop pulmonary hypertension (PH) and right heart failure. Impaired production of endogenous nitric oxide (NO) plays an important role in the pathogenesis of PF-associated PH. The NO signaling pathway involves activation of soluble guanylate cyclase (sGC) with subsequent generation of cGMP. We hypothesized that sGC might be involved in the pathogenesis of PF and that the NO-independent stimulation of sGC might attenuate PH and fibrotic changes in a clinically relevant mouse model of PF.

## Materials and methods

Male C57/BL6 mice (10-12 wks) were anesthetized, intubated, and subjected to intratracheal administration of bleomycin (0.5 U/kg) or saline. The animals were randomly assigned to gavage-feeding with the sGC stimulator riociguat (1, 3 or 10 mg/kg/day), the phosphodiesterase 5 (PDE5) inhibitor sildenafil (100 mg/kg/day), a combination of riociguat (1 mg/kg/day) and sildenafil (100 mg/kg/day), or vehicle alone for two weeks. Thereafter, the mice underwent transthoracic echocardiographic and invasive hemodynamic measurements. Hearts, lungs and blood were harvested. Two independent pathologists blindly measured a surface area involved by fibrosis and inflammation in hematoxylin and eosin stained lung sections.

## Results

Bleomycin-induced PH (an increase in the right ventricle systolic pressure and a decrease in the pulmonary acceleration time/ejection time ratio) and the right ventricular hypertrophy were attenuated by riociguat and the combination of riociguat and sildenafil to a greater extent than by sildenafil alone. In the vehicle-treated mice, fibrosis and inflammation diffusely involved lung parenchyma. Riociguat and the combination of riociguat and sildenafil but not sildenafil alone markedly ameliorated PF and inflammation that was mainly confined to subpleural areas and/or peripheral lung in a patchy distribution. Riociguat increased plasma cGMP concentration and also reduced mortality.

## Conclusion

Pharmacological stimulation of sGC with riociguat attenuates PF, PH, right ventricular hypertrophy and mortality in the bleomycin-exposed mice. This therapeutic approach appears to be superior to treatment with PDE5 inhibitor sildenafil. This is consistent with the mode of action: riociguat increases sGC activity in the absence of NO and also sensitizes sGC to low levels of endogenous NO, while PDE5 inhibitors prevent the degradation of cGMP and thus rely on a sufficient upstream input of NO. Stimulation of sGC might represent a new modality for treating PF and related conditions.

